# Emerging Roles of Dermal Fibroblasts in Hyperpigmentation and Hypopigmentation: A Review

**DOI:** 10.1111/jocd.16790

**Published:** 2025-01-08

**Authors:** Xingyue Gao, Wenzhong Xiang

**Affiliations:** ^1^ Department of Dermatology Hangzhou Third People's Hospital, Zhejiang Chinese Medical University Zhejiang Hangzhou China; ^2^ Department of Dermatology Hangzhou Third People's Hospital Zhejiang Hangzhou China

**Keywords:** cytokine, fibroblasts, melanocytes, pigmentation

## Abstract

**Background:**

Skin pigmentation disorders may increase patients' psychological burdens. Consequently, they are increasingly attracting attention. Dermal fibroblasts have been shown to regulate pigmentation by secreting soluble factors.

**Aim:**

This study aimed to summarize recent findings on the effects of dermal fibroblasts on hyperpigmentation and hypopigmentation, enabling the discovery of new therapeutic targets.

**Methods:**

PubMed was searched for literature on fibroblast factors, hyperpigmentation, and hypopigmentation, and a comprehensive summary and analysis were performed.

**Results:**

Fibroblasts secrete both cytokines that promote pigmentation, including stem cell factor (SCF) and keratinocyte growth factor (KGF), and small amounts of those that inhibit pigmentation, such as Dickkopf1 (DKK1) and transforming growth factor (TGF)‐β. Fibroblast‐derived extracellular matrix (ECM) can also affect melanocyte tyrosinase activity and the transfer of melanosomes. In hyperpigmentation disorders, such as melasma and solar lentigines, the secretion of pigmentation‐promoting factors increases, and the activity of key enzymes in melanin production is elevated. In hypopigmentation disorders, including vitiligo, the secretion of melanogenic factors decreases while the factors that inhibit pigmentation increase. Fibroblasts may serve as a new therapeutic target, providing new insights to precisely treat pigmentary disorders.

**Conclusions:**

Fibroblasts synthesize and secrete various cytokines and proteins that modify melanin synthesis and transfer through different signaling pathways, playing prominent roles in pigmentary skin disorders, such as photoaging, melasma, solar lentigo, and vitiligo.

## 1 Introduction

Attention to skin pigmentation disorders, including hyperpigmentation and hypopigmentation, has increased. These disorders may increase the psychological burdens of patients due to increased anxiety, diminished self‐esteem, and interpersonal problems, specifically patients with vitiligo [1, 2]. Melasma also negatively impacts patients' quality of life [3]. Additionally, the economic burden of treatment and the potential impact on professional and social lives further underline the importance of understanding and addressing these disorders. The cellular mechanisms of pigmentation disorders have been extensively studied, focusing primarily on keratinocytes and melanocytes, which play critical roles in pigmentation. However, the role of dermal fibroblasts, another critical skin cell type, has not received sufficient attention despite emerging evidence of their involvement in pigmentation regulation.

Dermal fibroblasts are a primary cellular components of the dermis. They are essential in wound healing and the secretion of the extracellular matrix (ECM) and cytokines. Additionally, they are involved in photoaging, skin homeostasis, morphogenesis, structural maintenance, immune surveillance, and in interactions with other cell types [4]. Recent studies have revealed that dermal fibroblasts regulate pigmentation by secreting soluble factors that influence melanocyte function through paracrine signaling. Key factors such as basic fibroblast growth factor (bFGF), hepatocyte growth factor (HGF), and endothelin‐1 (ET‐1) have been implicated in either promoting or inhibiting pigmentation.

This review summarizes recent findings regarding the effects of dermal fibroblasts on pigmentation, highlighting their roles in both hyperpigmentation and hypopigmentation. We hope to identify novel therapeutic targets for pigmentary disorders by shedding light on these mechanisms.

## 2 Materials and Methods

We comprehensively search PubMed for literature on fibroblast factors, hyperpigmentation, and hypopigmentation. Two authors reviewed the literature, screening for eligible studies, and summarized the findings.

## 3 Results and Discussion

### 3.1 Physiological Function of Dermal Fibroblasts

Similar to keratinocytes, fibroblasts release various cytokines to regulate melanocyte activity, such as stem cell factor (SCF), keratinocyte growth factor (KGF), hepatocyte growth factor (HGF), and basic fibroblast growth factor (bFGF) [5], which are known to promote the growth, survival, and melanogenesis of melanocytes. These paracrine agents bind to specific receptors expressed on melanocytes, accelerate signal transductions, and initiate melanin production. In addition, a few cytokines secreted by fibroblasts can also inhibit pigmentation.

SCF, which is synthesized by keratinocytes and fibroblasts, plays an important role in adjusting the life cycle of human melanocytes and maintaining melanocytes' survival. In an experimental study, human SCF remarkably increased the number of human melanocytes compared to murine SCF. Other studies indicated that the rise in SCF expression increased skin pigmentation. Soluble SCF can activate epidermal melanocytes by diffusing to the epidermis. SCF binds to c‐kit on melanocytes, further activating the extracellular signal‐regulated kinase (ERK) and phosphoinositide 3‐kinase (PI3K)/protein kinase B (Akt) signaling pathways, thereby promoting the activity and health of melanocytes [1, 6]. Other members in the mitogen‐activated protein kinase (MAPK) family can also be activated and alter the activity and function of the microphthalmia‐associated transcription factor (MITF). MITF is the most pivotal transcription factor regulating melanocytes [7].

KGF/FGF7 is a member of the fibroblast growth factor (FGF) family and a key regulatory factor for epithelial growth and differentiation. KGF induces tyrosinase expression in primary melanocytes; promotes the transfer of melanosomes from melanocytes to keratinocytes by binding and activating its tyrosine kinase receptor, KGFR; and facilitates the phagocytic process of keratinocytes [8, 9, 10]. The specific receptor KGFR is expressed on keratinocytes but not on melanocytes or fibroblasts [11]. KGF stimulates keratinocytes to secrete SCF and generates an indirect paracrine network connecting keratinocytes and fibroblasts, thereby controlling melanocyte function [5].

HGF is one of the melanogenic cytokines synthesized by fibroblasts. HGF enhances cell proliferation, motility, and melanogenesis by binding to the mesenchymal–epithelial transition factor (c‐met) on melanocytes and activating the MAPK and PI3K/Akt signaling pathway [1]. HGF plays a dual role in skin pigmentation. It also participates in losing the adhesion molecule E‐cadherin in melanocytes, which helps melanocytes separate from adjacent keratinocytes [12].

As an effective mitogen, bFGF stimulates melanocyte proliferation and promotes melanocyte migration. In previous studies, the c‐Jun N‐terminal kinase (JNK) pathway was found to be essential for regulating cell migration. The mechanism by which bFGF promotes melanocyte migration is related to the activation of the PI3K/Akt rac1‐FAK‐JNK and ERK signaling pathways.

In addition to the aforementioned cytokines, many fibroblast‐derived factors that can promote pigmentation. Secreted frizzled‐related protein 2 (sFRP2) is produced by fibroblasts, especially those exposed to UVB irradiation [13]; in a coculture experiment, it increased pigmentation in normal human melanocytes [13]. In melanocytes, sFRP2 acts as a melanogenic stimulator through β‐catenin signaling leading to the up‐regulation of MITF and TYR [9]. Wnt inhibitory factor‐1 (WIF‐1) can also promote melanin synthesis in melanocytes. Neuregulin1 (NRG1), a novel fibroblast‐derived neuroendocrine factor that regulates human skin pigmentation [14], increases the pigmentation and size of melanocytes and participates in determining the human skin color. NRG1 mediates melanogenesis via the PI3K/Akt pathway, increasing melanocyte proliferation, survival, and melanin synthesis [14]. NRG1 can also enhance the expression of melanocyte‐specific genes, such as *MITF*, *DCT*, *TYR*, and *c‐KIT* [14]. Other melanogenesis‐modulating factors, like stromal‐derived factor 1 (SDF1) and growth differentiation factor15 (GDF15), also increase skin pigmentation [15]. Endothelin 1 (EDN1), produced by fibroblasts, keratinocytes, and endothelial cells, activates the melanogenesis pathway by binding to the endothelin receptor B (EDNRB) on the surface of melanocytes.

Evidence suggests that fibroblasts and fibroblast‐derived ECM proteins influence melanocyte proliferation, apoptosis resistance, morphology, and melanogenesis activity [16, 17]. The ECM can also affect melanocyte tyrosinase activity and the transfer of melanosomes. Fibrins present in the ECM control the morphology of melanocytes.

As for the fibroblast‐derived cytokines that inhibit pigmentation, Dickkopf1 (DKK1) is the most studied. Fibroblasts' most obvious physiological effect on melanocytes is in palmoplantar skin, which is significantly less pigmented because fibroblasts in human palmoplantar skin express substantially higher levels of DKK1 [17]. DKK1 is a secreted Wnt antagonist [18]. It inhibits melanocyte proliferation and melanin synthesis by inhibiting the Wnt/β‐catenin/MITF pathway [18]. Furthermore, it can suppress Peroxisome Activator Receptor (PAR)‐2 expression in keratinocytes thus reducing melanin transfer. It inhibits the expression level of melanocyte‐specific markers including DCT, MART1, TYR, GP100/Pmel17, and MITF [18]. DKK1 suppresses the activity of tyrosinase by upregulating the expression of membrane‐associated transporter protein, leading to a decrease in melanogenesis [1]. In addition, similar to HGF, DKK1 is involved in reducing the expression of E‐cadherin [12].

Transforming growth factor (TGF)‐β is primarily secreted by fibroblasts and in small amounts by macrophages, which regulate macrophage function [7]. An experimental study has reported that TGF‐β inhibits cAMP/protein kinase A (PKA) signal transduction, induces GLI2, and subsequently suppresses MITF. Pleiotrophin (PTN) is a secreted heparin‐binding protein with diverse biological functions. PTN is expressed in melanocytes and fibroblasts of human skin. Studies using transgenic animals have shown that PTN may degrade MITF by activating melanocyte ERK1/2 and reducing melanin production. Fibroblast‐derived clusterin (CLU) negatively affects cutaneous pigmentation [15].

**FIGURE 1:**
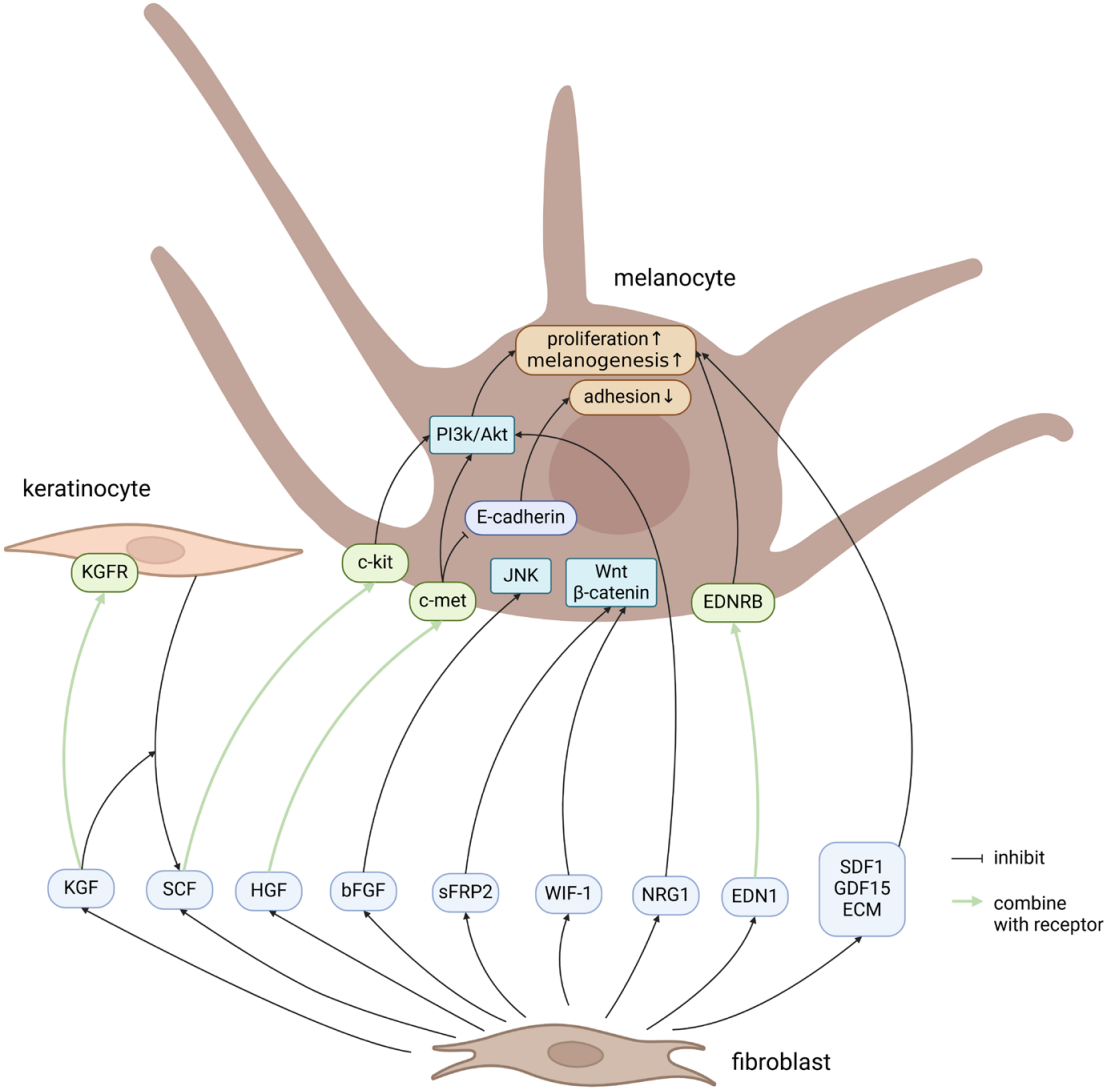
Cytokines secreted by fibroblasts that promote pigmentation. Akt, protein kinase B; bFGF, basic fibroblast growth factor; ECM, extracellular matrix; EDN1, Endothelin 1; EDNRB, endothelin receptor B; GDF15, growth differentiation factor15; HGF, hepatocyte growth factor; JNK, c‐Jun N‐terminal kinase; KGF, keratinocyte growth factor; KGFR, keratinocyte growth factor receptor; NRG1, Neuregulin1; PI3K, phosphoinositide 3‐kinase; SCF, stem cell factor; SDF1, stromal‐derived factor 1; sFRP2, secreted frizzled‐related protein 2; WIF‐1, Wnt inhibitory factor‐1.

**FIGURE 2:**
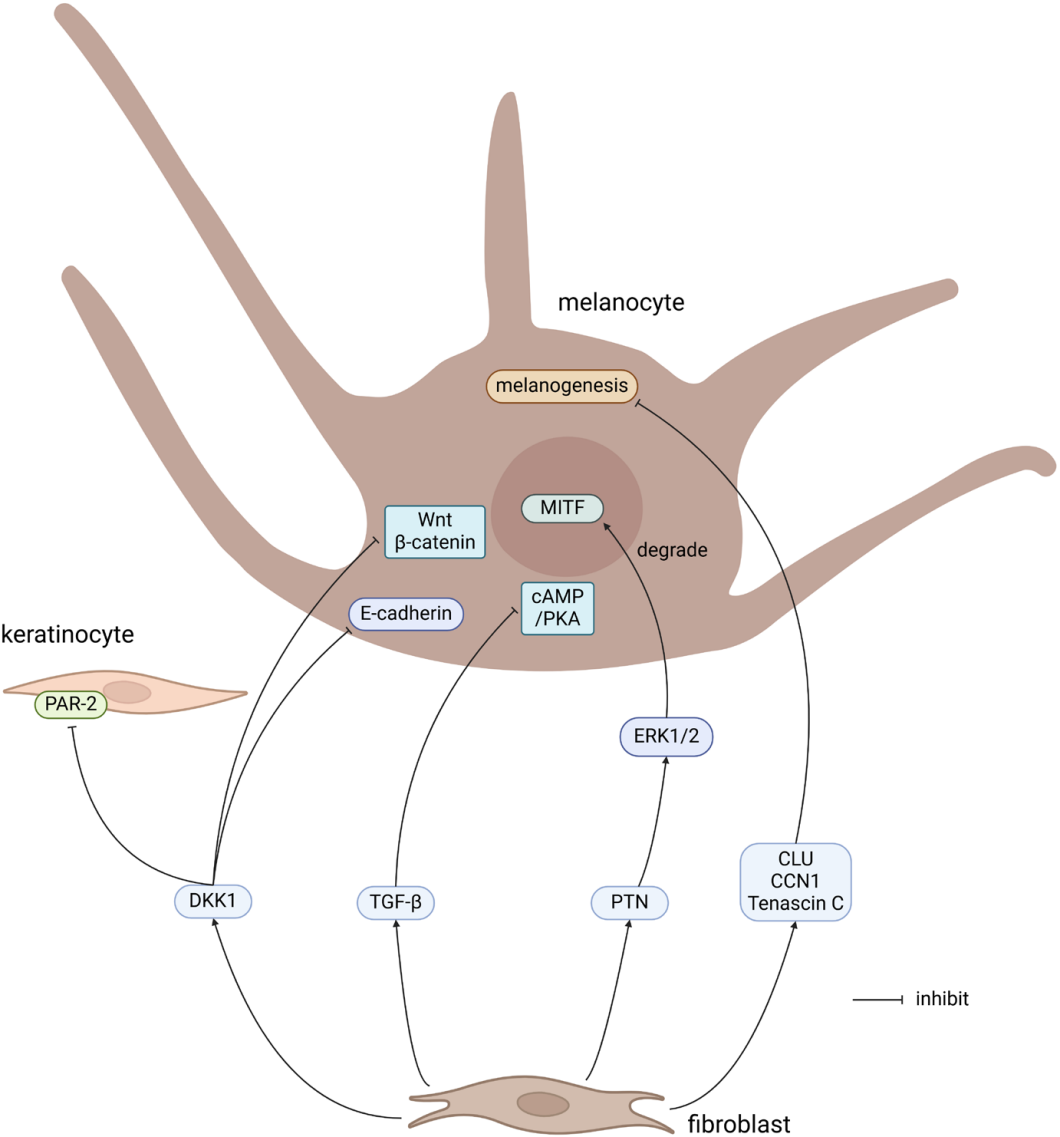
Cytokines secreted by fibroblasts that inhibit pigmentation. CLU, clusterin; DKK1, Dickkopf1; MITF, microphthalmia‐associated transcription factor; PAR‐2, Peroxisome Activator Receptor‐2; PKA, protein kinase A; PTN, Pleiotrophin; TGF‐β, Transforming growth factor‐β.

Table 1 and Figures 1, 2 summarize the cytokines secreted by fibroblasts. Biorender was used to create the figures in the study.

**TABLE 1:** Summary of cytokines secreted by fibroblasts.

Cytokines	Receptor and/or cell signaling pathways	Effect
SCF	c‐kit receptor, PI3k/Akt	Adjust the life cycle of human melanocytes and maintain the survival of melanocytes, alter the activity and function of MITF
KGF	KGFR (keratinocytes)	Promote the transfer of melanosomes from melanocytes to keratinocytes, facilitate the phagocytic process of keratinocytes, stimulate keratinocytes to secrete SCF
HGF	c‐met receptor, MAPK, PI3k/Akt	Enhance cell proliferation, motility, and melanogenesis, participate in the loss of the adhesion molecule E‐cadherin in melanocytes
bFGF	PI3k/Akt, Rac1‐FAK‐JNK, ERK	Stimulate melanocyte proliferation and promote melanocyte migration
sFRP2		Upregulate the expression of MITF and TYR
WIF‐1		Promote melanin synthesis in melanocytes
NRG1	PI3k/Akt	Increase the pigmentation and size of melanocytes, enhance the expression of melanocyte‐specific genes
SDF1		Modulate melanogenesis
GDF15		Modulate melanogenesis
EDN1	EDNRB	Activate the melanogenesis pathway
ECM proteins		Influence the proliferation, apoptosis resistance, morphology, and melanogenesis activity of melanocytes
DKK1	Wnt/β‐catenin	Inhibit melanocyte proliferation and melanin synthesis, suppress the expression of PAR‐2 in keratinocytes, inhibit the expression level of melanocytes‐specific markers including DCT, MART1, TYR, GP100/Pmel17 and MITF, suppresses the activity of tyrosinase, reduce the expression of E‐cadherin
TGF‐β	cAMP/PKA	Suppress MITF
PTN		Degrade MITF
CLU		Have a negative effect on cutaneous pigmentation
CCN1		Play an important role in the development of hyperpigmentation disorders
Tenascin C		Exacerbate melanocyte detachment

Abbreviations: Akt, protein kinase B; bFGF, basic fibroblast growth factor; CLU, clusterin; DKK1, Dickkopf1; ECM, extracellular matrix; EDN1, Endothelin 1; EDNRB, endothelin receptor B; GDF15, growth differentiation factor15; HGF, hepatocyte growth factor; JNK, c‐Jun N‐terminal kinase; KGF, keratinocyte growth factor; KGFR, keratinocyte growth factor receptor; MAPK, mitogen‐activated protein kinase; MITF, microphthalmia‐associated transcription factor; NRG1, Neuregulin1; PAR‐2, Peroxisome Activator Receptor‐2; PI3K, phosphoinositide 3‐kinase; PKA, protein kinase A; PTN, Pleiotrophin; SCF, stem cell factor; SDF1, stromal‐derived factor 1; sFRP2, secreted frizzled‐related protein 2; TGF‐β, Transforming growth factor‐β; WIF‐1, Wnt inhibitory factor‐1.

### 3.2 Rising Role of Fibroblasts in Pigmentary Disorders

Pigmentation, which occurs during the process of skin aging, is of concern to people. The most important senescent cells in skin‐aging pigmentation are fibroblasts and melanocytes. Senescent fibroblasts are the prominent participants in the sustained activation of melanocytes, which may lead to increased pigmentation in photoaging colored skin. The characteristic of pigmented skin with photoaging is the accumulation of aging fibroblasts [15, 19]. Skin exposure to ultraviolet radiation (UVR) can cause an increase in reactive oxygen species (ROS), thereby activating signaling pathways involving epidermal keratinocytes and dermal fibroblasts, as well as activating of inflammatory genes. For instance, PGF2α is produced by fibroblasts and keratinocytes, stimulates the formation of melanocyte dendrites and activates tyrosinase; however, it cannot promote proliferation.

Melasma is a common acquired symmetrical hyperpigmentation of melanin. It is now generally accepted that complex interactions between epidermal melanocytes, keratinocytes, dermal fibroblasts, and vascular endothelial cells cause melasma [3]. Current research indicates that melasma is characterized by the accumulation of aging cells in the dermis of the lesional skin, along with senescent fibroblasts, which may contribute to the increased pigmentation observed in melasma. Melasma exhibits distinct features of UV damage, including an increased number of senescent fibroblasts in the lesions, solar elastosis, basement membrane disruption, and increased epidermal pigmentation [15]. Photo‐damaged fibroblasts have melanin‐promoting functions and may take part in the pathogenesis of melasma. SCF in the dermis and the c‐kit receptor on melanocytes are the significant factors controlling pigmentation in patients with melasma [8, 20]. The expression of sFRP2 in melasma skin is upregulated, promoting pigmentation [9]. Although WIF‐1 is a cytokine that promotes pigmentation, as mentioned above, studies have revealed that decreased WIF‐1 expression is associated with skin pigmentation in patients with melasma.

Solar lentigo is characterized by pigmented skin lesions that occur in photo‐damaged areas, with the number and size of these lesions increasing with age. SCF, KGF, and HGF are the main fibroblast secretion factors that promote the occurrence of solar lentigo [8, 16]. sFRP2 is also found to be upregulated in the lesions of solar lentigo [9]. While solar lentigo is caused by UV exposure, senile lentigo results primarily due to skin aging. It is reported that senescent fibroblasts play a critical role in pigmentation related to aging [19]. These senescent fibroblasts stimulate pigmentation by upregulating the expression of melanogenesis regulators, such as MITF and tyrosinase, in melanocytes [19].

Acanthosis nigricans (AN) is a skin condition with varying etiologies, characterized by symmetrical, dark, rough, thickened, velvety plaques commonly found on the neck, armpits, elbow creases, inframammary folds, and groin area [21]. The pathophysiological mechanism of AN involves the proliferation of epidermal keratinocytes and dermal fibroblasts stimulated by multiple factors [21], indicating that fibroblasts play a role in the development and progression of AN.

Riehl melanosis was first reported in 1917 to describe typical pigmented lesions on the face and neck, mainly occurring after episodes of contact dermatitis. CCN1 (CYR61, cysteine‐rich 61) is a fibroblast‐derived paracrine mediator of melanogenesis, secreted under UVB irradiation. An in vitro cell study demonstrated that CCN1 may be involved in the development of hyperpigmentation disorders such as Riehl melanosis [22].

Dermatofibroma is a common benign skin nodule notable for the prominent pigmentation of the epidermis above the lesion, often accompanied by mild acanthosis. Café‐au‐lait macules are areas of skin with light to dark brown coloration, characterized by well‐defined borders and an absence of hair. In the pigmentation of the epidermis in dermatofibroma and café‐au‐lait macules, soluble SCF secreted by dermal fibroblasts activates melanocytes by diffusing into the epidermis.

Vitiligo is the most prevalent hypopigmentation disorder. It is characterized by well‐defined, variably shaped depigmented patches and plaques that appear on various parts of the body. Fibroblasts are involved in oxidative stress in vitiligo [12] and can mobilize immune cells by producing cytokines and regulating the immune response [5]. Increased oxidative stress accelerates cellular aging. Senescent fibroblasts lose their normal function, and the dysregulated secretion of growth factors leads to the disappearance of melanocytes. A recent study found that vitiligo involves of IFN‐γ responsive fibroblasts, which can recruit T cells [23]. This is significant for studying vitiligo pathogenesis. The cytokines secreted by fibroblasts have an essential impact on the pathogenesis of vitiligo. The expression levels of SCF in vitiligo lesions did not decrease; however, the expression of the SCF receptor c‐kit protein at the lesion margins decreased significantly [6, 8]. Purpura et al. [11] documented that fibroblasts within the lesions exhibit diminished melanosome transfer and lowered KGF mRNA expression in patients with vitiligo. Furthermore, in these patients, the expression levels of depigmentation‐inducing factors increase while those promoting pigmentation decrease [8]. Chen et al. [24] have demonstrated experimentally that the expression of DKK1 in the dermis of vitiligo lesions is increased. Compared to normal fibroblasts, vitiligo fibroblasts secrete higher levels of DKK1 and HGF, reducing the expression of the adhesion molecule E‐cadherin in melanocytes [12]. The reduction of E‐cadherin can lead to melanocytes detaching from the basement membrane, subsequently resulting in melanocyte death. Fibroblasts in vitiligo lesions secrete increased amounts of tenascin C, and its elevated levels in the dermis may promote or exacerbate melanocyte detachment. Collagen secreted by fibroblasts can also affect melanocytes. The study by Yokoi et al. [25] revealed that collagen production and antioxidative enzyme release increased in vitiligo fibroblasts, while collagen degeneration was inhibited. Furthermore, Bastonini et al. [9] demonstrated that in the lesions of patients with vitiligo, the expression of type IV collagen is reduced, inhibiting the adhesion and binding properties of melanocytes, possibly leading to melanocyte loss.

## 4 Conclusions

Recent studies demonstrated that fibroblasts influence pigmentation, and their impact on melanocyte activity and metabolism has attracted interest. Fibroblasts synthesize and secrete various cytokines and proteins, modifying melanin synthesis and transfer through different signaling pathways. Fibroblasts are involved in skin conditions, such as photoaging, melasma, solar lentigo, AN, and vitiligo, and can serve as a target for new therapeutic approaches, providing new insights for the precise treatment of pigmentary disorders.

## Author Contributions

Xingyue Gao was responsible for original draft writing and investigation. Wenzhong Xiang provided the conceptualization and reviewed the article.

## Ethics Statement

The authors have nothing to report.

## Conflicts of Interest

The authors declare no conflicts of interest.

## Data Availability

The data that support the findings of this study are available from the corresponding author upon reasonable request.
